# Workplace Violence Against Healthcare Workers in the Emergency Department at a Tertiary Academic Institute in Riyadh, Saudi Arabia: Patterns and Associated Factors

**DOI:** 10.3390/healthcare14193188

**Published:** 2026-09-27

**Authors:** Bandar M. Almutairi, Fatima Mohammed Halawi, Salman Nabeel Almane, Samu Abdulrahman Alzeer, Faris Jaber Alotaibi, Abdullah Mansour Almuneef, Mohammed Hamad Alhumud, Yasser A. Alaska

**Affiliations:** 1 College of Medicine, King Saud University, Riyadh 12372, Saudi Arabia; 2 Department of Family and Community Medicine, College of Medicine, King Saud University, Riyadh 12372, Saudi Arabia; 3 School of Public Health, Faculty of Medicine, Imperial College London, London W12 0BZ, UK; 4 Department of Emergency Medicine, College of Medicine, King Saud University, Riyadh 12372, Saudi Arabia

**Keywords:** workplace violence, healthcare workers, emergency department, verbal abuse, physical violence, Saudi Arabia

## Abstract

**Highlights:**

**What are the main findings?**

- In the previous 12 months, 8.8% (95% CI 5.5–13.9) of participating emergency department healthcare workers reported being physically attacked and 39.2% (95% CI 32.4–46.5) reported verbal abuse, most often by patients and their relatives; among the 74 respondents exposed to physical attack and/or verbal abuse, only 16 (21.6%, 95% CI 13.8–32.3) completed a formal institutional report.
- Routine direct physical contact with patients was independently associated with exposure to physical attack and/or verbal abuse in this sample (adjusted OR = 3.27); nurses and the combined “Other” group had lower adjusted odds than physicians when the remaining model variables were held constant. Because the groups differ substantially in routine patient contact, this is a conditional contrast rather than a general statement about occupational risk.

**What are the implications of the main findings?**

- In this cross-sectional sample, exposure was associated with routine direct physical contact with patients and with professional groups; estimates for gender and years of experience were imprecise and neither support nor exclude an association.
- Infrequent formal institutional reporting and dissatisfaction with incident handling call for non-punitive, accessible reporting systems, de-escalation training, and visible organizational support.

**Abstract:**

Background/Objectives: Workplace violence (WPV) against healthcare workers (HCWs), especially in emergency departments (EDs), is a growing concern both globally and in Saudi Arabia. This study aimed to describe the proportion of participating staff reporting physical attack and verbal abuse, identify the patterns and associated factors of these experiences in the emergency department at King Saud University Medical City (KSUMC), and assess healthcare workers’ perceptions of existing preventive measures. Methods: A cross-sectional study was conducted at KSUMC, Riyadh. Using convenience sampling, staff with direct patient or family interaction were asked to complete an adapted version of the World Health Organization (WHO) Workplace Violence in the Health Sector Questionnaire. Descriptive statistics, Wilson 95% confidence intervals (CIs) for proportions, and logistic regression were used to describe responses and to examine factors associated with exposure. The adapted instrument retained the incident-specific modules on physical attack and verbal abuse; the modules on threats, bullying or mobbing, sexual harassment and racial harassment were omitted, so the primary outcome represents physical attack and/or verbal abuse rather than the full workplace-violence spectrum. Results: Of 181 participants, 8.8% (95% CI 5.5–13.9) reported being physically attacked and 39.2% (95% CI 32.4–46.5) reported verbal abuse in the previous 12 months; 74 (40.9%, 95% CI 34.0–48.2) reported either form. Among these 74 exposed respondents, 16 (21.6%, 95% CI 13.8–32.3) completed a formal institutional report (Occurrence Variance Report or incident/accident form); among the 102 respondents with a reportable incident, 31 (30.4%, 95% CI 22.3–39.9) indicated on a summary item that they had reported an incident by any route. In multivariable analysis, routine direct physical contact with patients or clients was independently associated with exposure (adjusted OR = 3.27, 95% CI 1.36–7.89), while nurses and other professional groups had lower adjusted odds than physicians. Conclusions: Self-reported exposure to physical attack and/or verbal abuse was common among participating KSUMC ED staff, particularly those with routine direct physical contact with patients or clients. Because participants were recruited by convenience at a single center, these figures describe the participating sample rather than population prevalence. Formal institutional reporting was infrequent, supporting attention to non-punitive reporting systems, de-escalation training, and qualitative follow-up.

## 1. Introduction

According to the World Health Organization (WHO), violence is described as “the deliberate use of physical force or power, threatened or actual, against oneself, another person, or against a group or community, that has consequences or has a high probability of resulting in injury, death, mental distress, maldevelopment, or deprivation” [1], and according to the National Institute for Occupational Safety and Health (NIOSH), workplace violence (WPV) is defined as “violent acts, including physical assaults and threats of assaults, directed toward persons at work or on duty” [2]. In healthcare settings, WPV is conventionally understood as a spectrum rather than a single behavior, encompassing physical assault, verbal abuse, threats, bullying or mobbing, and sexual harassment, and is most often initiated by patients or their accompanying relatives rather than by co-workers or intruders [3].

WPV against healthcare providers is a growing concern worldwide, particularly in emergency department (ED) settings, where the fast-paced and high-stress environment, frequently compounded by severe overcrowding and system delays, increases the risk of violent incidents [4,5]. International studies have reported a high prevalence of WPV against healthcare workers (HCWs); for example, studies in China [6], Turkey [7], and the United States reported prevalence rates of 79.39%, 97.4%, and 70.6%, respectively [8]. However, this wide variance in reported prevalence across different countries is largely attributable to significant methodological differences. Specifically, estimates are heavily influenced by the distinct clinical settings studied, variations in professional composition, differing operational definitions of violence, inconsistent recall periods, and varied sampling and questionnaire administration methods [9].

In Saudi Arabia, studies have found a high prevalence of WPV [4,10,11]. A study conducted in Riyadh reported that 59% of HCWs in EDs experienced violence, predominantly verbal or physical assaults by patients or their families [10]. Another study conducted in the Eastern Region reported that 37.1% of HCWs experienced verbal abuse and 11.4% experienced or witnessed physical abuse, predominantly by patients’ families [11].

Studies indicate that nursing staff and less experienced HCWs are at a higher risk of experiencing violence, and violent behaviors are more commonly associated with male and older patients [12]. Beyond these demographic trends, the literature identifies specific occupational and operational variables as key risk factors for workplace violence. Characteristics such as the worker’s professional group, level of clinical experience, frequency of direct patient contact, and the undertaking of night shifts are recognized predictors of exposure to aggressive behavior in emergency settings [6]. Furthermore, an institution’s safety culture, specifically whether staff are actively encouraged to report incidents, plays a crucial role in accurately capturing prevalence and effectively managing WPV [5]. WPV against HCWs is associated with an increased risk of psychological consequences such as depression and post-traumatic stress disorder (PTSD) [13,14]. Additionally, WPV can also decrease occupational performance and increase the risk of burnout [14]. Mechanistically, exposure to workplace aggression acts as an overwhelming job demand that rapidly depletes a healthcare worker’s emotional and cognitive resources, activating a cycle of emotional exhaustion and depersonalization [13,14]. These consequences are thought to arise through several linked pathways: severe psychological depletion, the development of post-traumatic stress symptoms that persist beyond the incident itself, and avoidance behaviors such as the intention to leave the profession, each of which may in turn affect the continuity and quality of care delivered [13,14].

Proposed interventions include communication skills training for healthcare workers, clear reporting systems, and workplace safety training [5,12]. In Saudi Arabia, Ministry of Health regulations make verbal and physical abuse of healthcare workers punishable, underscoring the importance of protecting healthcare personnel [15].

While previous Saudi studies have documented the prevalence of violence in various hospital settings, they have largely relied on heterogeneous definitions and focused on descriptive outcomes. Considerably less is known about the nature, circumstances, and institutional responses surrounding these events. Focusing specifically on an academic tertiary emergency department provides essential context regarding a highly stressed environment heavily reliant on emergency medicine physicians in training. Investigating this specific setting adds new knowledge by addressing how unique operational pressures combined with a high concentration of medical trainees who may have varying levels of de-escalation experience influence the dynamics and risks of workplace aggression [16].

Emergency departments internationally share features that have been proposed as contributors to patient- and relative-initiated aggression, including emotional distress, unmet expectations and system delays. None of these mechanisms was measured in the present study, and the design is not able to test them. What this study offers is a detailed, denominator-explicit description of exposure, circumstances and reporting behavior in one academic tertiary emergency department, which may be useful for comparison with similar settings. Therefore, this study aimed to describe the proportion of participating staff reporting physical attack and verbal abuse, identify the patterns and associated factors of these experiences in the emergency department at King Saud University Medical City (KSUMC), and assess healthcare workers’ perceptions of existing preventive measures.

## 2. Materials and Methods

### 2.1. Participants and Study Design

A cross-sectional study was conducted to investigate the occurrence and nature of workplace violence among healthcare professionals, reported in accordance with the STROBE statement for cross-sectional studies [17] (Supplementary Table S1). The study was situated at KSUMC in Riyadh, Saudi Arabia, and the data were collected from June 2025 to September 2025. This four-month window corresponded to the period covered by the approved study protocol and to the availability of the research team for on-site recruitment; recruitment ended once the planned visits had been completed rather than when a fixed sample size had been reached.

The target population consisted of healthcare workers, including physicians, nurses, and administrative support staff, currently working in the emergency department with direct patient or family interaction. Participants were recruited from the emergency department at KSUMC by convenience sampling across 50 recruitment visits; at each visit all individuals working at the time were invited to take part. Visit dates were generated prior to data collection using a computer-generated random date selector. To ensure proportionate coverage across work schedules, recruitment visits were distributed across morning, evening, and overnight shifts on both weekdays and weekends. Healthcare workers were approached individually by members of the research team in staff-only areas; supervisors and managers were not present during recruitment, so participation was voluntary and free from workplace pressure. The numbers of employees approached, eligible, or declining to participate were not recorded by field personnel, precluding exact response rate calculations. Participants completed the questionnaire through an anonymous online link that could be opened at a time and place of the respondent’s choosing; supervisors and managers could not identify who took part. To prevent duplicate participation, the survey included a question indicating whether the respondent had previously completed it, and those who had already participated were thanked and asked not to fill it in again. The sample size was initially determined using the Raosoft calculator (Raosoft, Inc., Seattle, WA, USA); based on a total population of 365 healthcare workers and a population proportion of 50%, a target of 188 participants was calculated to maintain a 95% confidence level and a 5% margin of error. Ultimately, a total of 181 completed surveys were collected and included in the final analysis, slightly below the calculated target. This calculation was intended for the estimation of a single proportion and assumes probability-based sampling; however, participants were recruited using convenience sampling. The sample size calculation does not eliminate the selection bias inherent in convenience recruitment. Accordingly, the observed proportions are interpreted as proportions among participating respondents rather than as population prevalence estimates. The 74 outcome events available for the multivariable model correspond to approximately nine events per estimated coefficient, slightly below a commonly cited heuristic threshold of ten. Participants were eligible if they were working in the KSUMC emergency department during the recruitment period, belonged to any professional group, including physicians of any grade, nurses, allied health professionals, pharmacists, technical staff, security and support staff, and administrative staff, had a role involving interaction with patients or their relatives, and provided informed consent. No minimum length of employment was required; therefore, newly appointed staff and residents rotating through the department were eligible. Staff whose roles involved no patient or family interaction, staff not working in the department during the recruitment period, those who declined participation, and duplicate responses were excluded. Nine respondents reported characteristics that appeared inconsistent with these criteria, and the two groups differ. Three answered “No” to an item asking whether they worked at King Khalid University Hospital (KKUH), the name used interchangeably with King Saud University Medical City for this site. On their remaining responses, all three were full-time emergency department clinicians who reported interaction with patients and routine direct physical contact with them. Because the survey was anonymous and collected no identifiers, this discrepancy could not be resolved against employment records; the three responses were retained, and a sensitivity analysis excluding them is reported below. Six respondents (3.3%) reported no routine interaction with patients or clients; five of these worked in support roles and one in a technical role, and they therefore fall outside the patient-interaction criterion. Because of the questionnaire’s skip logic, these six were not shown the follow-up item on routine direct physical contact, and the multivariable model is accordingly a complete-case analysis of the remaining 175 respondents. A sensitivity analysis excluding all nine respondents (*n* = 172) is reported in Supplementary Table S9, and a further analysis coding the six structurally missing responses as “No” is reported in Supplementary Table S10; neither materially changed the findings.

### 2.2. Data Collection Procedure

Data were collected from June to September 2025, which represented the predefined data-collection period for the study. Data were collected using an anonymous online questionnaire. Staff were approached individually by a member of the research team and completed the questionnaire through an online link at a time and place of their choosing. Supervisors and managers were not involved in recruitment and could not identify who participated. No employee numbers, email addresses, or IP addresses were collected. Two optional free-text fields, for a name and a mobile number, appeared on the final page of the questionnaire; no respondent completed either field, and neither was included in the analytic dataset or used in any analysis. To prevent duplicate participation, the survey included a question asking whether the respondent had previously completed it; respondents who indicated that they had already participated were asked not to complete it again.

Participants were recruited using convenience sampling during recruitment visits conducted on randomly selected days. Recruitment covered weekdays and weekends, as well as morning, evening, and night shifts, to include staff working across different schedules. The numbers of individuals approached, eligible, refusing participation, or unavailable during recruitment were not recorded; therefore, a response rate could not be calculated, and the representativeness of the sample could not be determined. Although the initial sample size was estimated using the Raosoft sample size calculator, with a 95% confidence level and a 5% margin of error, resulting in a target sample of 188 participants, a total of 181 participants were ultimately included in the study. The use of convenience sampling remained subject to potential selection bias.

### 2.3. Study Instrument

The study used an adapted English version of the ILO/ICN/WHO/PSI Workplace Violence in the Health Sector Country Case Study Questionnaire [18]. The administered questionnaire contained 72 question prompts, including matrix questions comprising multiple individual items. Conditional skip logic was applied, such that not all questions were applicable to every participant. The questionnaire included personal and workplace characteristics; experience of physical workplace violence and verbal abuse during the previous 12 months; the circumstances, perpetrators, consequences, and reporting of these incidents; employer policies and workplace measures; and respondents’ views on the helpfulness of preventive measures. The original incident-specific modules assessing bullying/mobbing, sexual harassment, racial harassment, and threats were omitted; however, these forms of workplace violence remained included in questions assessing employer policies. Study-specific items on physician grade and Emergency Medicine board certification were added. The questionnaire was administered in English, the standard operational and clinical documentation language of the department. Before full deployment the adapted instrument was piloted among 10 emergency department healthcare professionals of varying roles and nationalities to confirm item readability and face validity; only minor formatting adjustments followed, and no items were reworded, so the pilot responses were not retained in the analysis. Participants were told that the questionnaire would take approximately 4–6 min to complete. The administered questionnaire is provided as Supplementary File S1. Most items were categorical, using yes/no or multiple-response formats. Items assessing the psychological impact of an incident used a five-point response scale ranging from “Not at all” to “Extremely”, while perceived helpfulness of preventive measures was rated on a four-point scale coded 0 (not at all helpful) to 3 (very helpful) and summarized as item-level means with standard deviations. Items were analyzed individually; no total score, subscale score, or validated cut-off was applied, and responses were therefore reported and interpreted at the item level.

In addition to the sections of the instrument, the following participant and occupational characteristics were recorded: age group, gender, marital status, and nationality; professional group and, for physicians, grade and emergency medicine board certification; migration to the country of work and time since migration; years of experience in the health sector; employing institution; employment status (full-time, part-time, or temporary/casual); shift work and night work between 18:00 and 07:00; routine interaction with patients or relatives and routine direct physical contact with patients; the age groups and genders of patients cared for; work setting and staffing levels; degree of worry about workplace violence; and awareness of, and encouragement to use, violence-reporting procedures.

### 2.4. Statistical Analysis

All analyses were performed using Stata version 17 (StataCorp, College Station, TX, USA). Categorical variables are summarized as frequencies and percentages. For every conditional item the denominator is the number of respondents who reached that item under the questionnaire’s skip logic and is stated explicitly in the corresponding table row. For multiple-response items, percentages are calculated among all respondents entering that branch and therefore need not sum to 100%. Missing values were not imputed.

The principal outcomes—physical attack and verbal abuse in the previous 12 months—are summarized as proportions among participating respondents, with Wilson score 95% confidence intervals (CIs); Wilson intervals were preferred to normal-approximation intervals because several subgroup denominators were small.

Reporting behavior was analyzed among respondents with a reportable incident, defined as having experienced physical attack, experienced verbal abuse, and/or witnessed physical violence in the previous 12 months.

Items assessing reactions to an incident were recorded on a five-point scale from “Not at all” to “Extremely” and were analyzed individually. Perceived helpfulness of preventive measures was rated on a four-point scale coded 0 (not at all helpful) to 3 (very helpful) and summarized as item-level means with standard deviations.

Associations between individual characteristics and exposure to physical attack and/or verbal abuse were examined using binary logistic regression. Univariable logistic regression was performed for each candidate factor, and a multivariable model was then fitted with six variables entered simultaneously. These variables were selected on conceptual grounds.

Six respondents who reported no interaction with patients or clients were not shown the follow-up item on routine direct physical contact, because of the questionnaire’s skip logic; their responses on this item are structurally missing rather than missing at random. The multivariable model is therefore a complete-case analysis restricted to the 175 respondents who answered this item. The univariable models reported alongside the adjusted estimates were fitted within the same 175-respondent sample.

Because the multivariable model estimated eight coefficients from 74 outcome events and several covariate categories were sparse—most notably the combined “Other” professional group, which contained four outcome events—the model was refitted using Firth penalized-likelihood logistic regression as a small-sample robustness check [19], with penalized profile-likelihood confidence intervals (Supplementary Table S11). Multicollinearity was assessed using variance inflation factors (VIFs). Model discrimination was assessed using the area under the receiver operating characteristic curve (AUC) with its 95% CI. Model calibration was assessed with the Hosmer–Lemeshow test. Regression coefficients with their standard errors are reported in Supplementary Table S8. Two sensitivity analyses were performed: the model was refitted after excluding the nine respondents whose reported characteristics were inconsistent with the eligibility criteria (Supplementary Table S9) and after coding the six structurally missing patient-contact responses as “No” (Supplementary Table S10). A two-sided *p*-value < 0.05 was considered statistically significant.

### 2.5. Ethical Approval

Ethical approval was obtained from the Institutional Review Board (IRB) at King Saud University, College of Medicine. Informed consent was obtained from all participants prior to enrollment, and strict measures were implemented to maintain data confidentiality and anonymity in accordance with the Declaration of Helsinki. Because the questionnaire asked about injury, psychological reactions, fear of negative consequences, and possible disciplinary action, additional safeguards applied: participation was voluntary and anonymous; no employee numbers, email addresses, or IP addresses were collected; two optional free-text fields for a name and a mobile number were offered on the final page but were left blank by every respondent and were excluded from the analytic dataset; respondents could complete the survey privately at a time of their choosing; and managers could not determine who participated. Results are reported in aggregate only, and small professional groups were combined so that individuals could not be identified. Participants were not provided with specific information about occupational health or counselling services as part of the study. The complete administered questionnaire, including the item on disciplinary action in connection with reporting, was submitted to and approved by the IRB as part of the study protocol, and the full item wording is reproduced in Supplementary File S1. The application described the instrument in full but did not name reporting-related disciplinary consequences as a separate study objective, and the corresponding finding is reported as a signal requiring independent investigation rather than as evidence of an established institutional practice. Generative artificial intelligence (Claude Opus 4.7, Anthropic) was used to edit selected passages of the manuscript for clarity and organization. It was not used to generate or analyze data, select statistical methods, interpret findings, or formulate the study conclusions. All AI-edited text was critically reviewed, verified, and edited by the authors, who take full responsibility for the content of the publication.

## 3. Results

Of the 181 healthcare workers recruited from the emergency department at King Saud University Medical City (KSUMC), all 181 provided analyzable responses to the core exposure items; the analytic sample for the multivariable regression was 175. Full item-level distributions for the more granular questionnaire sections are provided in Supplementary Tables S2–S7.

### 3.1. Participant and Workplace Characteristics

Table 1 summarizes the sociodemographic characteristics of the 181 participants. The majority were female (95/181, 52.5%) and under 30 years of age (102/181, 56.4%); most were single (110/181, 60.8%) and Saudi nationals (117/181, 64.6%).

All participants worked in the emergency department. By professional group, 70 (38.7%) were physicians, 87 (48.1%) were nurses, and 24 (13.3%) belonged to the combined “Other” category, which comprised support staff, allied health professionals, pharmacists, medical interns, technical staff, a certified registered nurse, a patient-care technician and one administrator. Among the 69 physicians who answered the item on physician grade, 25 (36.2%) were junior residents, 15 (21.7%) senior residents, 15 (21.7%) consultants, 6 (8.7%) assistant consultants, 4 (5.8%) associate consultants and 4 (5.8%) staff physicians. Emergency medicine board certification, recorded only for the 70 physicians, was held by 29 (41.4%).

Most participants had five years of experience or less (118/181, 65.2%), full-time (179/181, 98.9%), in shifts (175/181, 96.7%), and at some time between 18:00 and 07:00 (162/181, 89.5%). Routine direct physical contact with patients or clients was reported by 125 of the 175 respondents shown the conditional item (71.4%). Concern about workplace violence was mixed: 57/181 (31.5%) were moderately worried and 48/181 (26.5%) were not worried at all. Reporting procedures were said to exist by 163/181 (90.1%), and among the 162 who answered the follow-up item, 126 (77.8%) knew how to use them; 131/181 (72.4%) reported being encouraged to report violence. Full workplace-characteristic distributions are provided in Supplementary Table S2.

Nine respondents reported characteristics that appeared inconsistent with the eligibility criteria. Six (3.3%) reported no routine interaction with patients or clients; five of these worked in support roles and one in a technical role, and they therefore fall outside the patient-interaction criterion. Three answered “No” to an item asking whether they worked at King Khalid University Hospital (KKUH), the name used interchangeably with King Saud University Medical City for this site; on their remaining responses all three were full-time emergency department clinicians reporting interaction with patients and routine direct physical contact. Because the survey was anonymous and collected no identifiers, these discrepancies could not be resolved against employment records; all nine responses were retained, and a sensitivity analysis excluding them is reported in Supplementary Table S9.

### 3.2. Exposure to Physical Attack and Verbal Abuse

Table 2 summarizes self-reported exposure in the previous 12 months. These figures describe proportions among participating respondents recruited by convenience at a single center and should not be read as population prevalence estimates. They do not cover threats, bullying or mobbing, sexual harassment or racial harassment, for which the corresponding questionnaire modules were not administered.

Among the 16 respondents physically attacked, all described the most recent incident as physical violence without a weapon and 14 (87.5%) considered it typical of their workplace. Among the 71 verbally abused, this occurred “sometimes” for 48 (67.6%), “once” for 12 (16.9%) and “all the time” for 11 (15.5%). Among the 71 who witnessed physical violence, this occurred two to four times for 34 (47.9%), once for 22 (31.0%), five to ten times for 12 (16.9%), several times a month for 2 (2.8%) and about once a week for 1 (1.4%).

### 3.3. Perpetrators, Location and Circumstances

For the 16 physical-attack incidents, the reported perpetrator was a patient or client for 14 (87.5%), a relative of a patient or client for 8 (50.0%), a staff member for 5 (31.3%), an external colleague or worker for 2 (12.5%) and management or a supervisor for 1 (6.3%), multiple responses being permitted; 13 (81.3%) occurred inside the health facility, 2 (12.5%) at a patient’s home and 1 (6.3%) outside the workplace.

For the 71 verbal-abuse incidents, the perpetrator was a relative of a patient or client for 43 (60.6%), a patient or client for 37 (52.1%), a staff member for 16 (22.5%), management or a supervisor for 9 (12.7%), an external colleague or worker for 5 (7.0%) and a member of the general public for 3 (4.2%); 66 (93.0%) occurred inside the health facility, 3 (4.2%) at a patient’s home and 2 (2.8%) outside the workplace. A colleague or a manager was named as a perpetrator of physical or verbal aggression by 24 of the 74 exposed respondents (32.4%, 95% CI 22.9–43.7), a source with different prevention implications from patient-initiated aggression. Full circumstantial detail, including time of day, day of week and item-level response actions, is provided in Supplementary Tables S3 and S4.

### 3.4. Reporting Behavior and Institutional Response

For this section a reportable incident is defined as having experienced physical attack, experienced verbal abuse, and/or witnessed physical violence in the previous 12 months; 102 of 181 respondents (56.4%, 95% CI 49.1–63.4) met this definition. Table 3 presents reporting and institutional-response measures with the denominator stated for each.

The figure of 35/181 (19.3%) takes the entire study sample as its denominator and includes respondents with no reportable incident and therefore no opportunity to report one; it is not a reporting rate. Among respondents with at least one reportable incident, 31/102 (30.4%, 95% CI 22.3–39.9) answered “yes” to the summary reporting item, indicating that they had reported an incident by any route, and the proportion was similar among those exposed in their own right (23/74, 31.1%) and among witnesses only (8/28, 28.6%). Formal institutional reporting specifically was recorded by 16 of the 74 exposed respondents (21.6%, 95% CI 13.8–32.3).

Four respondents answered “yes” to the summary reporting item without a corresponding reportable event recorded elsewhere in their responses; all four were nurses. This discrepancy could not be resolved from the anonymous dataset. It does not affect the reportable-incident denominator of 102 or the exposed denominator of 74, both of which derive from the exposure and witnessing items rather than from the summary item, but it should be borne in mind when interpreting that item.

Of the 35 respondents who answered “yes” to the summary reporting item, 33 answered a follow-up item asking whether they had been disciplined in connection with reporting, and 16 of these 33 (48.5%, 95% CI 32.5–64.8) indicated that they had. The administered item asked explicitly whether the respondent had been disciplined for reporting an incident (Supplementary File S1), so it refers to action directed at the person who made the report rather than at the person responsible for the incident. The finding nonetheless rests on a single, non-validated item, answered by a modest number of respondents, administered in English to a multilingual workforce. Cross-tabulation shows that only 7 of these 16 respondents also recorded having completed a formal institutional report elsewhere in the questionnaire, the item having followed the summary reporting question rather than the formal-report question. It is therefore presented as a signal warranting further investigation rather than as an established feature of departmental practice.

### 3.5. Self-Reported Reactions to Physical Attack and Verbal Abuse

Table 4 and Table 5 summarize self-reported reactions among respondents who reported physical attack and verbal abuse, respectively, in the previous 12 months. The figures describe the distribution of responses within each subgroup.

Participants who reported physical attack (*n* = 16) and verbal abuse (*n* = 71) rated five items describing possible reactions—how bothered they were, intrusive memories, avoidance, hypervigilance and a sense that everything was an effort—on a five-point scale from “Not at all” to “Extremely”. These are responses to individual, non-validated items; no composite or subscale score was computed, no diagnostic threshold was applied, and the cross-sectional design cannot establish that the reactions described were caused by the reported incident. They should not be read as an assessment of psychological morbidity, post-traumatic stress disorder, burnout or long-term occupational impact.

As threshold summaries only, and not as validated severity classifications, 12 of the 16 respondents who experienced physical attack (75.0%, 95% CI 50.5–89.8) endorsed at least one item at “Moderately” or above and 5 (31.3%, 95% CI 14.2–55.6) at “Quite a bit” or above. Among the 71 who experienced verbal abuse, 57 (80.3%, 95% CI 69.6–87.9) endorsed at least one item at “Moderately” or above, 34 (47.9%, 95% CI 36.7–59.3) at “Quite a bit” or above, and 3 (4.2%, 95% CI 1.4–11.7) endorsed no item at all.

### 3.6. Employer Policies and Workplace Measures

Most participants reported that their employer had developed policies on health and safety (134/181, 74.0%), physical workplace violence (128/181, 70.7%), sexual harassment (112/181, 61.9%), verbal abuse (111/181, 61.3%), threats (108/181, 59.7%), bullying or mobbing (99/181, 54.7%) and racial harassment (89/181, 49.2%); between 20.4% and 36.5% answered “do not know” across these items. The most frequently reported practical measures were security measures (155/181, 85.6%), restricted public access (82/181, 45.3%), improved surroundings (73/181, 40.3%) and patient protocols (73/181, 40.3%), while 5/181 (2.8%) reported none of the listed measures. Reported workplace changes over the previous two years included increased staff numbers (28/181, 15.5%) and staff cuts (28/181, 15.5%) in equal proportion; because multiple responses were permitted these may reflect different units or different times. A large proportion, 74/181 (40.9%), did not know whether changes had occurred. Perceptions of the impact of any changes were divided, with 39/181 (21.5%) reporting that the staff work situation had worsened and 37/181 (20.4%) that it had improved, while 63/181 (34.8%) did not know. Full distributions are provided in Supplementary Table S5.

### 3.7. Perceived Helpfulness of Preventive Measures

Table 6 shows the perceived helpfulness of 13 workplace safety and violence-prevention measures, rated on a 0–3 scale and ranked by mean rating. Security measures received the highest mean rating and restricting the exchange of money at the workplace the lowest, indicating that respondents rated visible, immediate protective measures more highly than organizational or scheduling measures. The four-category distributions underlying each mean are given in Supplementary Table S6.

### 3.8. Factors Associated with Exposure to Physical Attack and/or Verbal Abuse

In the 175-respondent analytic sample, 74 respondents were exposed (58 verbal abuse only, 3 physical attack only, 13 both). Table 7 presents univariable and adjusted odds ratios together with the number of outcome events in every covariate category.

After adjustment, professional group and routine direct physical contact remained independently associated with exposure. Respondents with routine direct physical contact with patients had more than three times the adjusted odds of exposure (adjusted OR 3.27, 95% CI 1.36–7.89), while nurses (adjusted OR 0.37, 95% CI 0.15–0.91) and the combined “Other” group (adjusted OR 0.22, 95% CI 0.06–0.82) had lower adjusted odds than physicians. Gender, work experience, night-shift work and encouragement to report were not significantly associated with exposure; with 74 events and eight estimated coefficients these estimates are imprecise and are better read as inconclusive than as evidence of no association.

The adjusted odds ratio for nurses (0.37) is lower than the univariable estimate obtained in the same sample (0.75). This is consistent with confounding by routine direct physical contact: nurses had a markedly higher prevalence of such contact than physicians (82/87, 94.3% versus 32/70, 45.7%; “Other” 11/18, 61.1%; χ^2^(2) = 45.83, *p* < 0.001), and contact was itself associated with higher odds of exposure. In the unadjusted comparison the high contact rate among nurses offsets part of their lower odds of exposure relative to physicians; once contact is held constant, the nurse–physician contrast increases in magnitude. With a confidence interval of 0.15 to 0.91 the adjusted estimate nonetheless remains imprecise, and the “Other” category, which contains only four outcome events across eight occupations, should be interpreted with particular caution. Firth penalized-likelihood estimation produced attenuated but consistent estimates (Supplementary Table S11): routine direct physical contact 3.04 (penalized profile-likelihood 95% CI 1.33–7.34), nurses 0.40 (0.16–0.93) and the combined “Other” group 0.26 (0.07–0.81) relative to physicians. Attenuation was greatest for the “Other” group, as expected given its four outcome events, but all three intervals continued to exclude unity and the direction and statistical interpretation of every estimate were unchanged, indicating that the main estimates are not artefacts of sparse-data bias. Model discrimination was modest (AUC = 0.657, 95% CI 0.576–0.738) and the included variables accounted for a small proportion of the variation in exposure (Nagelkerke *R*^2^ = 0.118). The model is accordingly presented as exploratory and associational; it characterises associations within this sample and is not a predictive model.

## 4. Discussion

Workplace violence represents a persistent and complex challenge within emergency care settings, with substantial implications for healthcare workers’ safety and organizational performance. The findings of the present study contribute to the growing body of evidence highlighting the vulnerability of emergency department staff to both physical and non-physical forms of aggression. The patterns observed in this study are consistent with structural and environmental features of emergency medicine, including high patient acuity, emotional volatility, and operational pressures [4].

Three levels of inference should be distinguished in what follows. The first comprises findings supported directly by these data: the proportions of respondents reporting physical attack and verbal abuse, the reported perpetrators and locations, the denominator-specific reporting proportions, and the adjusted associations with routine direct physical contact and professional group. The second comprises interpretations drawn from previous research that are consistent with, but not tested by, these data: the contribution of workload, waiting times, overcrowding, family expectations, clinical seniority and organizational culture, none of which was measured here. The third comprises questions these data raise but cannot answer: whether reporting-related disciplinary action occurs and how often, why formal documentation was so much less common than informal disclosure, and whether any preventive measure rated highly by respondents would in fact reduce incidents. Statements below should be read according to which category they fall into; the qualification is not repeated at each occurrence.

Although exposure in this sample did not differ significantly by basic demographic characteristics such as gender, physician work experience differences may contribute to the lower incidents of WPV, work experience was not associated with exposure in either the univariable or the adjusted model.

Less experienced practitioners may not yet have developed communication and de-escalation skills that can mitigate conflict during high-tension encounters. This is in line with observations from Saudi and international research where younger or less experienced staff reported higher incidences of workplace violence due to their frequent involvement in direct patient interactions [20].

Occupational characteristics emerged as the factors with the strongest association with violence exposure. Healthcare workers reporting routine direct physical contact with patients or clients had the highest adjusted odds of exposure. This association may reflect the greater frequency and intensity of close interpersonal interactions involved in direct patient care, which may create more opportunities for conflict or confrontation. This finding is consistent with previous research in Saudi emergency departments, where frontline clinical roles and close patient proximity have been associated with workplace violence [10]. Similarly, studies conducted in Riyadh have reported high levels of physical and verbal violence in EDs, particularly in settings characterized by high patient loads and prolonged waiting times [11]. Physician grade was recorded only for physicians and was not included in the model, so this remains an untested explanation rather than a demonstrated mechanism.

Importantly, the patterns of alleged perpetrators in the current study, predominantly patients and their relatives, reinforce existing evidence that violence in healthcare settings often stems from emotional distress, frustration with care processes, or unmet expectations. Research conducted in Riyadh EDs also found that relatives were a major source of both verbal and physical aggression, with a significant proportion of incidents occurring during high-pressure moments of care [11]. This pattern suggests that interventions must address not only patient behavior but also family expectations and communication strategies, especially in culturally complex clinical environments. The broader healthcare literature suggests that workplace violence is experienced by staff across diverse backgrounds, and organizational culture may modulate reporting and response behaviors. Previous work has indicated that isolation or lack of institutional support can intensify the psychological burden of violence, even if it does not directly increase exposure rates [4].

A central finding concerns reporting behavior. Despite formal mechanisms being in place, many healthcare workers either failed to report violent incidents or relied on informal channels. This phenomenon has been widely documented in the literature, where perceived inefficacy of reporting systems, fear of retaliation, and a belief that incidents are part of “the job” discourage formal documentation of violence [10,20]. Such underreporting undermines institutional capacity to quantify and respond to workplace safety challenges effectively. Enhancing organizational trust and establishing non-punitive reporting cultures are therefore critical to improving both data quality and intervention strategies.

One observation warrants particular attention. Of the 33 respondents who answered the relevant item, 16 indicated that they had been disciplined in connection with reporting an incident. The item asked explicitly whether the respondent had been disciplined for reporting an incident (Supplementary File S1), so it refers to action directed at the person who made the report rather than at the aggressor. Such a response may discourage subsequent reporting and may help explain the limited number of formal reports. The item was nonetheless single and unvalidated and was answered by a small number of respondents, so the finding is presented as a signal warranting further investigation rather than as an established feature of the department.

Taken together, the associations observed here involved occupational rather than demographic characteristics. Consistent with previous research [4,10,11,18], effective prevention and mitigation require multifaceted approaches that integrate policy enforcement, communication training, de-escalation strategies, and visible administrative support. Strengthening these aspects can help reduce the burden of violence on healthcare workers, improve psychological well-being, and contribute to safer, higher-quality patient care in emergency settings.

Among the 16 participants who reported a physical attack, the most frequently endorsed response to four of the five impact items was “Moderately”, with intrusive memories, avoidance, hypervigilance, and a sense that everything was an effort each endorsed to some degree. These are individual self-reported items rather than a validated instrument, the subgroup is small, and the cross-sectional design cannot establish that the incident caused the reactions described. This is consistent with the previous literature suggesting that workplace violence adversely affects psychological well-being and job performance among healthcare workers, particularly in high-stress environments such as emergency departments [5,6].

Among participants who reported verbal abuse, responses were distributed across the full range of each item, with being “super-alert” or watchful and feeling that everything was an effort most commonly endorsed as “A little bit” or “Moderately.” Previous studies have linked verbal abuse with burnout, job dissatisfaction, and psychological strain [6,11]. Although recurrent exposure has been hypothesized to contribute to cumulative psychological effects, the present cross-sectional, item-level data cannot establish this relationship and are therefore reported descriptively.

Verbal abuse was considerably more common than physical attack in this sample, consistent with findings from Saudi Arabia and internationally, where verbal aggression is frequently reported as the predominant form of workplace violence in healthcare settings, particularly in emergency departments [4,10,21]. Patients and their relatives were identified as the primary perpetrators, which is consistent with existing evidence suggesting that factors such as emotional distress, overcrowding, and dissatisfaction with care may contribute to aggressive behaviors [6,11].

Despite these frequent exposures, reporting behavior was limited, and the route mattered. Among the 74 exposed respondents, 34 (45.9%) told a senior staff member and 20 (27.0%) told a colleague, whereas only 16 (21.6%) completed a formal institutional report and 29 (39.2%) took no action after the most recent incident. Informal disclosure was therefore roughly twice as common as formal documentation. This observation is consistent with previous research indicating that workplace violence is often underreported because of perceived ineffectiveness of reporting systems, fear of negative consequences, and lack of organizational support [7,11].

Furthermore, satisfaction with how incidents were managed was generally low, highlighting deficiencies in organizational response and enforcement mechanisms. Previous studies have similarly emphasized that inadequate institutional policies and lack of accountability may contribute to the persistence and underreporting of workplace violence [7].

Importantly, most participants believed that incidents of verbal abuse could have been prevented, indicating that workplace violence is not an inevitable aspect of emergency care but a modifiable one. The previous literature has examined strategies such as communication skills training, de-escalation strategies, and improvements in workplace systems [7,8]. However, the present survey assessed participants’ beliefs about preventability and perceptions of helpfulness rather than intervention effectiveness. These findings therefore reflect participant perceptions and priorities rather than evidence that any specific intervention would reduce workplace violence in this setting. Additionally, enforcement of national regulations that criminalize violence against healthcare workers may play a crucial role in reducing such incidents [15].

Overall, the findings highlight that both physical and verbal workplace violence have significant psychological implications. While physical violence may lead to more immediate effects, verbal abuse, due to its higher frequency and repetitive nature, may result in a cumulative psychological burden over time. Addressing both forms of violence is therefore essential to improve healthcare workers’ well-being, enhance workplace safety, and maintain the quality of patient care.

Most participants were aware that their workplace had policies addressing health and safety, physical violence, verbal abuse, sexual harassment, threats, and bullying. This suggests institutional recognition of workplace violence as a safety issue; however, the presence of a written policy does not by itself establish effective implementation. Previous research has likewise identified formal policies as an important initial component of violence prevention [10].

Security measures such as guards, alarms, and portable telephones were the most commonly reported practical interventions (85.6%). Organizational and patient-oriented measures—including public-access restrictions, patient protocols and screening, training, and increased staffing—were reported less often. This pattern suggests greater reliance on security-based approaches than on broader organizational and patient-oriented strategies. Prior research has associated organizational approaches with improved care and lower violence risk [22].

Reported workplace changes during the previous two years included both increased staffing and staff cuts (15.5% each). Because respondents could select multiple options, these findings may reflect changes in different units or at different times rather than a single organization-wide trend. Notably, 40.9% did not know whether changes had occurred, suggesting that organizational changes may not have been consistently communicated to staff.

Perceptions of the impact of workplace changes were divided: similar proportions reported that the staff work situation had worsened (21.5%) or improved (20.4%). These mixed responses may reflect variation in how changes affected workload, staffing, or teamwork across roles and units.

Healthcare workers rated security measures as the most helpful intervention, suggesting a preference for visible, immediate protection. Environmental measures, including restricted public access and improved surroundings, were also viewed favorably, as were patient protocols and screening. Organizational measures, such as increasing staff numbers and reducing periods of working alone, were generally rated moderately to very helpful, and training was also viewed positively. These findings are broadly consistent with prior research in which enhanced security, increased staffing, and training were among the most frequently proposed preventive measures [4].

In the adjusted analysis, routine direct physical contact with patients or clients showed the strongest association with reported violence exposure, with more than a three-fold increase in the adjusted odds. Professional groups were also associated with exposure, with nurses and other professional groups having lower adjusted odds than physicians. Gender and work experience were not significantly associated with exposure in this model; however, given the number of outcome events and the width of the confidence intervals, these estimates should be interpreted as imprecise rather than as evidence of no association. Overall, these findings suggest that occupational context may be relevant to exposure in this sample, but they should not be interpreted as establishing a general occupational pattern. They are partly consistent with previous studies in Saudi Arabia, in which profession was independently associated with violence exposure, although those studies also identified gender as an independent factor [10,16].

The professional-group estimates are conditional contrasts: they compare respondents in different professional groups who are alike on gender, work experience, night-shift work, routine direct physical contact and encouragement to report. This qualification is material here, because the groups differ markedly on one of those variables. Routine direct physical contact was reported by 94.3% of nurses, 45.7% of physicians and 61.1% of the combined “Other” group (χ^2^(2) = 45.83, *p* < 0.001), so overlap between the groups on a covariate strongly associated with exposure is limited, and the adjusted contrasts rest on a comparatively small number of directly comparable respondents. The “Other” category further combines eight occupations with different responsibilities and levels of patient exposure and contains only four outcome events. These estimates should therefore not be read as indicating that physicians are at greater risk of workplace violence than nurses in general. The unadjusted exposure proportions—48.6% of physicians, 41.4% of nurses and 22.2% of the “Other” group—are the appropriate basis for describing the group differences actually observed, and the difference between physicians and nurses in that comparison is small.

Overall, these results support prioritizing prevention strategies for healthcare workers whose duties involve routine direct physical contact with patients.

### 4.1. Theoretical and Practical Implications

Theoretically, these findings suggest that task-related characteristics may be relevant to understanding workplace-violence exposure in emergency care. Within this single-center sample, routine direct physical contact with patients or clients was associated with exposure, while the professional group also remained associated after adjustment. The relatively high proportion of physicians who were junior residents provides one possible explanation for the physician-group finding, but physician grade was not included in the model and this explanation remains untested. Practically, the findings suggest that staff whose duties involve close physical proximity to patients may warrant particular attention in prevention planning. De-escalation and communication training may be considered for frontline staff, while reporting systems may benefit from greater accessibility and reassurance that reporting will not result in negative consequences. However, because this survey measured perceived helpfulness rather than intervention effectiveness, these recommendations should be regarded as priorities for further evaluation rather than demonstrated solutions.

### 4.2. Strengths and Limitations

This study has several limitations. The use of convenience sampling at a single tertiary academic center limits the generalizability of the findings to other settings. Reliance on self-reported data is subject to recall and social-desirability bias, particularly for incidents that occurred up to 12 months before the survey. The cross-sectional design precludes causal inference regarding the associations identified, and the modest number of physical violence events (*n* = 16) limits the precision of the estimates for that outcome. The multivariable model estimated eight coefficients from 74 outcome events, slightly fewer than ten events per estimated parameter, so the adjusted associations—particularly the wide confidence intervals for professional group—should be interpreted with caution. Nonetheless, the use of an internationally established instrument, the WHO Workplace Violence in the Health Sector Questionnaire and the inclusion of multiple professional groups strengthen the internal validity of the findings. Because participation was voluntary and recruitment used convenience sampling at a single center, the reported figures represent proportions within this sample rather than population prevalence estimates and may be subject to selection and non-response bias. Several further limitations should be acknowledged. The planned sample of 188 was not achieved, and because the numbers approached, found ineligible, or declining were not recorded, the response rate and the extent of non-response bias are unknown; participation may have differed systematically by shift, professional group, or personal experience of violence. Six respondents in support and technical roles reported no routine interaction with patients or clients and therefore fell outside the stated eligibility criterion; because the questionnaire’s skip logic left their responses on routine direct physical contact structurally missing, they were excluded from the multivariable model rather than coded as a measured negative. A sensitivity analysis excluding these respondents together with the three who answered “No” to the site item did not materially change the findings. The questionnaire was administered in English only to a multinational workforce; although it was piloted for readability among 10 departmental staff, no formal linguistic or psychometric validation was undertaken in this setting, so some items, particularly those concerning psychological reactions and disciplinary consequences, may have been interpreted inconsistently. Exposure was self-reported over a 12-month recall window and is subject to misclassification. The composite outcome was dominated by verbal abuse (58 of 74 events), so the adjusted associations describe verbal abuse more than physical violence, and the heterogeneous “Other” professional category, together with several sparse professional subgroups, limits the interpretability of the corresponding odds ratios. The adapted instrument omitted the original incident-specific modules on threats, bullying or mobbing, sexual harassment and racial harassment; these categories were retained only in the items on employer policies, which record the existence of a policy rather than personal exposure. The primary outcome therefore captures physical attack and/or verbal abuse rather than the full spectrum of workplace violence described in the Introduction, and the figure of 40.9% will underestimate the broader burden of workplace violence in this department; the magnitude of that underestimation cannot be determined from these data. Finally, the survey assessed perceived helpfulness of preventive measures and cannot evaluate whether any intervention reduces incidents.

## 5. Conclusions

This study describes patterns of workplace violence (WPV) among healthcare workers in the emergency department (ED) at King Saud University Medical City (KSUMC). Self-reported exposure was common: 8.8% of participants reported physical attacks and 39.2% reported verbal abuse within the previous 12 months, with 40.9% reporting either form. Because the modules on threats, bullying or mobbing, sexual harassment and racial harassment were not administered, this figure represents physical attack and/or verbal abuse rather than workplace violence as a whole. These figures represent proportions within a single-center convenience sample and should not be interpreted as population prevalence estimates or generalized to Saudi healthcare workers. Consistent with the international and local literature, patients and their relatives were identified as the primary aggressors, and incidents occurred predominantly within the clinical setting.

A central observation is that routine direct physical contact with patients or clients was associated with reported violence exposure in this sample. This association should be interpreted as exploratory and does not establish that direct physical contact causes violence; the cross-sectional design precludes causal inference. While institutional policies and reporting mechanisms are in place, the study highlights a gap between policy existence and effective implementation, characterized by infrequent formal reporting and dissatisfaction with how incidents are managed. Only 16 of the 74 exposed respondents (21.6%) completed a formal institutional report, while 31 of the 102 respondents with a reportable incident (30.4%) indicated that they had reported an incident by any route.

Participants who experienced violence endorsed individual items describing heightened alertness, intrusive memories, avoidance, and a sense that everything was an effort; these were not assessed with a validated instrument and are reported descriptively rather than as measures of psychological morbidity or of long-term effects on well-being and performance. Although participants perceived security-based measures as particularly helpful, these findings reflect participant preferences rather than evidence of intervention effectiveness. A multifaceted approach incorporating security, organizational policies, reporting processes, and staff support may therefore warrant further evaluation.

Based on participants’ reported preferences and experiences, non-punitive and accessible reporting systems, together with de-escalation and communication training for frontline staff, may represent priorities for further evaluation at KSUMC. Organizational approaches that promote psychological safety and transparent communication may also warrant consideration, although their effectiveness was not evaluated in the present study. Qualitative research is also warranted to better understand staff experiences and to involve them in designing preventive measures.

## Figures and Tables

**Table 1:** Sociodemographic characteristics of the studied healthcare workers (*n* = 181).

Characteristic	Category	*n*	%
Gender	Male	86	47.5
	Female	95	52.5
Age (years)	<30	102	56.4
	30–39	53	29.3
	40–49	16	8.8
	50+	10	5.5
Marital status	Single	110	60.8
	Married	69	38.1
	Separated/divorced	1	0.6
	Widow/widower	1	0.6
Nationality	Saudi	117	64.6
	Non-Saudi	64	35.4

Data are frequency (%). The denominator for all rows is 181.

**Table 2:** Self-reported exposure to physical attack and verbal abuse in the previous 12 months (*n* = 181).

Outcome	*n*/*N*	%	95% CI
Physically attacked	16/181	8.8	5.5–13.9
Verbally abused	71/181	39.2	32.4–46.5
Physically attacked and/or verbally abused	74/181	40.9	34.0–48.2
Verbal abuse only	58/181	32.0	25.7–39.2
Physical attack only	3/181	1.7	0.6–4.8
Both forms	13/181	7.2	4.2–11.9
Witnessed physical violence	71/181	39.2	32.4–46.5
Experienced and/or witnessed ≥1 reportable incident	102/181	56.4	49.1–63.4

Data are *n*/*N* (%), where *n* is the number of respondents reporting the outcome and *N* is the denominator (181 for all rows). Wilson score 95% CIs.

**Table 3:** Reporting of workplace violence and institutional response.

Measure (Denominator)	*n*/*N*	%	95% CI
Reported an incident by any route (summary item), all respondents (*N* = 181)—descriptive only, not a reporting rate	35/181	19.3	14.2–25.7
Reported an incident by any route (summary item), respondents with a reportable incident (*N* = 102)	31/102	30.4	22.3–39.9
Among respondents exposed in their own right (*N* = 74)	23/74	31.1	21.7–42.3
Among witnesses only (*N* = 28)	8/28	28.6	15.3–47.1
Completed a formal institutional report (OVR or incident/accident form), among exposed respondents (*N* = 74)	16/74	21.6	13.8–32.3
OVR completed, among those physically attacked (*N* = 16)	6/16	37.5	18.5–61.4
Incident/accident form completed, among those verbally abused (*N* = 71)	12/71	16.9	9.9–27.3
Told a senior staff member, among exposed respondents (*N* = 74)	34/74	45.9	35.1–57.2
Told a colleague, among exposed respondents (*N* = 74)	20/74	27.0	18.2–38.1
Pursued prosecution, among exposed respondents (*N* = 74)	2/74	2.7	0.7–9.3
Took no action after the most recent incident, among exposed respondents (*N* = 74)	29/74	39.2	28.9–50.6
Disciplined in connection with reporting, among those answering the item (*N* = 33)	16/33	48.5	32.5–64.8

Data are *n*/*N* (%), where *n* is the number of respondents endorsing the measure and *N* is the denominator for that row, stated in parentheses in the first column. Denominators differ between rows because of the questionnaire’s skip logic and are not interchangeable. Wilson score 95% CIs. OVR, Occurrence Variance Report. Reporting routes are not mutually exclusive. The summary reporting item asked whether the respondent had reported an incident of workplace violence by any route and is not equivalent to completion of a formal institutional report.

**Table 4:** Self-reported reactions to physical attack (*n* = 16).

Item	Not at All	A Little Bit	Moderately	Quite a Bit	Extremely
Bothered by the experience	4 (25.0)	2 (12.5)	7 (43.8)	2 (12.5)	1 (6.3)
Repeated, disturbing memories	5 (31.3)	3 (18.8)	5 (31.3)	2 (12.5)	1 (6.3)
Avoiding thinking or talking about it	5 (31.3)	3 (18.8)	6 (37.5)	1 (6.3)	1 (6.3)
“Super-alert” or watchful	1 (6.3)	5 (31.3)	7 (43.8)	2 (12.5)	1 (6.3)
Everything felt an effort	3 (18.8)	5 (31.3)	6 (37.5)	1 (6.3)	1 (6.3)

Data are frequency (%). Denominator 16 for all rows.

**Table 5:** Self-reported reactions to verbal abuse (*n* = 71).

Item	Not at All	A Little Bit	Moderately	Quite a Bit	Extremely
Bothered by the experience	6 (8.5)	20 (28.2)	19 (26.8)	18 (25.4)	8 (11.3)
Repeated, disturbing memories	24 (33.8)	22 (31.0)	14 (19.7)	8 (11.3)	3 (4.2)
Avoiding thinking or talking about it	23 (32.4)	18 (25.4)	17 (23.9)	6 (8.5)	7 (9.9)
“Super-alert” or watchful	10 (14.1)	23 (32.4)	20 (28.2)	12 (16.9)	6 (8.5)
Everything felt an effort	12 (16.9)	20 (28.2)	22 (31.0)	11 (15.5)	6 (8.5)

Data are frequency (%). Denominator 71 for all rows.

**Table 6:** Perceived helpfulness of workplace violence prevention measures (*n* = 181).

Measure	Mean	SD
Security measures	2.54	0.85
Improve surroundings	2.36	0.88
Restrict public access	2.35	0.90
Training	2.30	0.86
Patient protocols	2.26	0.90
Patient screening	2.23	0.90
Reduced periods of working alone	2.19	0.93
Human resource development	2.16	0.91
Increased staff numbers	2.14	0.94
Check-in procedures for staff	2.10	0.97
Special equipment or clothing	1.91	1.04
Changed shifts or rotas	1.83	1.05
Restrict exchange of money at the workplace	1.73	1.08

Responses were coded 0 = not at all helpful, 1 = little, 2 = moderate, 3 = very helpful. *n* = 181 for every item.

**Table 7:** Univariable and multivariable logistic regression of factors associated with exposure to physical attack and/or verbal abuse (*n* = 175; 74 outcome events).

Variable	*n*	Events (%)	Univariable OR (95% CI)	*p*	Adjusted OR (95% CI)	*p*
Gender						
Male (ref)	81	32 (39.5)	1.00	—	1.00	—
Female	94	42 (44.7)	1.24 (0.68–2.26)	0.490	1.50 (0.74–3.03)	0.258
Professional group						
Physician (ref)	70	34 (48.6)	1.00	—	1.00	—
Nurse	87	36 (41.4)	0.75 (0.40–1.41)	0.368	0.37 (0.15–0.91)	0.031
Other	18	4 (22.2)	0.30 (0.09–1.01)	0.052	0.22 (0.06–0.82)	0.023
Work experience						
≤5 years (ref)	115	47 (40.9)	1.00	—	1.00	—
6–10 years	17	6 (35.3)	0.79 (0.27–2.28)	0.662	0.94 (0.31–2.82)	0.910
>10 years	43	21 (48.8)	1.38 (0.68–2.79)	0.369	1.62 (0.76–3.44)	0.209
Night-shift work, 18:00–07:00						
No (ref)	18	6 (33.3)	1.00	—	1.00	—
Yes	157	68 (43.3)	1.53 (0.55–4.28)	0.420	1.23 (0.41–3.68)	0.705
Routine direct physical contact						
No (ref)	50	15 (30.0)	1.00	—	1.00	—
Yes	125	59 (47.2)	2.09 (1.04–4.20)	0.039	3.27 (1.36–7.89)	0.008
Encouragement to report						
No (ref)	49	24 (49.0)	1.00	—	1.00	—
Yes	126	50 (39.7)	0.69 (0.35–1.33)	0.265	0.76 (0.36–1.59)	0.466

OR, odds ratio; CI, confidence interval; ref, reference category. Univariable models were fitted in the same 175-respondent sample as the multivariable model. Model summary: likelihood-ratio *χ*^2^(8) = 16.02, *p* = 0.042; McFadden pseudo-*R*^2^ = 0.067; Cox and Snell *R*^2^ = 0.088; Nagelkerke *R*^2^ = 0.118. Discrimination: AUC = 0.657 (95% CI 0.576–0.738). Calibration: Hosmer–Lemeshow *χ*^2^(7) = 0.59, *p* = 0.999. Variance inflation factors ranged from 1.06 to 1.95.

## Data Availability

The data presented in this study are available from the corresponding author on reasonable request, subject to approval by the King Saud University Institutional Review Board and a data-sharing agreement, to safeguard participant confidentiality.

## Supplementary Materials

The following supporting information can be downloaded at https://www.mdpi.com/article/10.3390/healthcare14193188/s1, Table S1: STROBE checklist for cross-sectional studies; Table S2: Workplace characteristics of participating healthcare workers; Table S3: Characteristics, consequences and responses to the most recent physical-violence incident; Table S4: Characteristics, consequences and responses to the most recent verbal-abuse incident; Table S5: Employer policies, workplace measures and reported changes; Table S6: Perceived helpfulness of preventive measures, full response distributions; Table S7: Exposure by covariate category in the analytic sample; Table S8: Main model, complete-case sample; Table S9: Sensitivity analysis excluding 9 inconsistent respondents; Table S10: Sensitivity analysis with missing routine-contact responses recoded to “No”; Table S11: Firth penalized-likelihood logistic regression, complete-case sample; File S1: Administered questionnaire.

## Abbreviations

The following abbreviations are used in this manuscript:

CI	Confidence interval
CRN	Certified Registered Nurse
ED	Emergency department
HCW	Healthcare worker
IRB	Institutional Review Board
KSUMC	King Saud University Medical City
NIOSH	National Institute for Occupational Safety and Health
OR	Odds ratio
OVR	Occurrence variance report
PTSD	Post-traumatic stress disorder
SD	Standard deviation
WHO	World Health Organization
WPV	Workplace violence
