# Supplementary material for: Workplace Violence Against Healthcare Workers in the Emergency Department at a Tertiary Academic Institute in Riyadh, Saudi Arabia: Patterns and Associated Factors
Source: Healthcare (Basel). 2026 Sep 27;14(19):3188. doi: 10.3390/healthcare14193188 (PMC13665011; doi:10.3390/healthcare14193188)
Supplement: Supplementary file 1 [file healthcare-14-03188-s001.zip › healthcare-4507128-supplementary.pdf]

Supplementary Material

Contents

Table S1. STROBE checklist for cross-sectional studies
Table S2. Workplace characteristics of participating healthcare workers
Table S3. Characteristics, consequences and responses to the most recent physical-violence incident
Table S4. Characteristics, consequences and responses to the most recent verbal-abuse incident
Table S5. Employer policies, workplace measures and reported changes
Table S6. Perceived helpfulness of preventive measures, full response distributions
Table S7. Exposure by covariate category in the analytic sample
Table S8. Main model, complete-case sample (n = 175; 74 events).
Table S9. Sensitivity analysis excluding 9 inconsistent respondents (n = 172; 74 events).
Table S10. Sensitivity analysis with missing routine-contact responses recoded to "No" (n = 181; 74 events).
Table S11. Firth penalized-likelihood logistic regression, complete-case sample (n = 175; 74 events).
Supplementary File S1. Administered questionnaire

Table S1. STROBE checklist for cross-sectional studies

	Item No	Recommendation	Reported in
**Title and abstract**	1	(*a*) Indicate the study's design with a commonly used term in the title or the abstract	Title; Abstract (Methods)
		(*b*) Provide in the abstract an informative and balanced summary of what was done and what was found	Abstract
**Introduction**			
Background/rationale	2	Explain the scientific background and rationale for the investigation being reported	Section 1, paragraphs 1–6
Objectives	3	State specific objectives, including any prespecified hypotheses	Section 1, final paragraph; Abstract
**Methods**			
Study design	4	Present key elements of study design early in the paper	Section 2.1; Abstract (Methods)
Setting	5	Describe the setting, locations, and relevant dates, including periods of recruitment, exposure, follow-up, and data collection	Section 2.1 (KKUH emergency department; June–September 2025; 50 recruitment visits)
Participants	6	(*a*) Give the eligibility criteria, and the sources and methods of selection of participants	Section 2.1
Variables	7	Clearly define all outcomes, exposures, predictors, potential confounders, and effect modifiers. Give diagnostic criteria, if applicable	Sections 2.3 and 2.4
Data sources/ measurement	8*	For each variable of interest, give sources of data and details of methods of assessment (measurement). Describe comparability of assessment methods if there is more than one group	Section 2.3; Supplementary File S5
Bias	9	Describe any efforts to address potential sources of bias	Sections 2.1, 2.2 and 4.2
Study size	10	Explain how the study size was arrived at	Section 2.1 (Raosoft; 188 planned, 181 obtained)
Quantitative variables	11	Explain how quantitative variables were handled in the analyses. If applicable, describe which groupings were chosen and why	Section 2.3 (age, experience and staffing recorded in categories)
Statistical methods	12	(*a*) Describe all statistical methods, including those used to control for confounding	Section 2.4
		(*b*) Describe any methods used to examine subgroups and interactions	Not applicable; no subgroup or interaction analyses were performed
		(*c*) Explain how missing data were addressed	Section 2.4 (complete-case analysis, n = 175)
		(*d*) If applicable, describe analytical methods taking account of sampling strategy	Section 2.1 (convenience sampling; no design weights applied)
		(*e*) Describe any sensitivity analyses	Section 2.1; Supplementary Tables S3 and S4
**Results**			

Participants	13*	(a) Report numbers of individuals at each stage of study—eg numbers potentially eligible, examined for eligibility, confirmed eligible, included in the study, completing follow-up, and analysed	Sections 2.1 and 3.1 (181 responses analysed; numbers approached not recorded)
		(b) Give reasons for non-participation at each stage	Sections 2.1 and 4.2 (response rate could not be determined)
		(c) Consider use of a flow diagram	Not applicable; numbers approached were not recorded
Descriptive data	14*	(a) Give characteristics of study participants (eg demographic, clinical, social) and information on exposures and potential confounders	Section 3.1; Tables 1 and 2
		(b) Indicate number of participants with missing data for each variable of interest	Section 2.4; footnote to Table 8
Outcome data	15*	Report numbers of outcome events or summary measures	Sections 3.2, 3.4 and 3.8; Tables 3 and 5
Main results	16	(*a*) Give unadjusted estimates and, if applicable, confounder-adjusted estimates and their precision (eg, 95% confidence interval). Make clear which confounders were adjusted for and why they were included	Section 3.8; Table 8; Supplementary Table S2
		(*b*) Report category boundaries when continuous variables were categorized	Section 3.8; Table 8
		(*c*) If relevant, consider translating estimates of relative risk into absolute risk for a meaningful time period	Not applicable; odds ratios were not converted to absolute risks
Other analyses	17	Report other analyses done—eg analyses of subgroups and interactions, and sensitivity analyses	Section 2.1; Supplementary Tables S3 and S4
		**Discussion**	
Key results	18	Summarise key results with reference to study objectives	Section 4, paragraphs 1–4
Limitations	19	Discuss limitations of the study, taking into account sources of potential bias or imprecision. Discuss both direction and magnitude of any potential bias	Section 4.2
Interpretation	20	Give a cautious overall interpretation of results considering objectives, limitations, multiplicity of analyses, results from similar studies, and other relevant evidence	Sections 4 and 4.1
Generalisability	21	Discuss the generalisability (external validity) of the study results	Sections 4.2 and 5; Abstract (Conclusions)
		**Other information**	
Funding	22	Give the source of funding and the role of the funders for the present study and, if applicable, for the original study on which the present article is based	Funding statement (no external funding)

*Give information separately for exposed and unexposed groups.

Note: An Explanation and Elaboration article discusses each checklist item and gives methodological background and published examples of transparent reporting. The STROBE checklist is best used in conjunction with this article (freely available on the Web sites of PLoS Medicine at http://www.plosmedicine.org/, Annals of Internal Medicine at http://www.annals.org/, and Epidemiology at http://www.epidem.com/). Information on the STROBE Initiative is available at www.strobe-statement.org.

Table S2. Workplace characteristics of participating healthcare workers (n = 181)

Characteristic	n	%	Denominator (N)
**Professional group**			
Physician	70	38.7	181
Nurse	87	48.1	181
Other healthcare or support professional	24	13.3	181
Composition of the "Other" group			
Support staff (kitchen, maintenance, security)	7	3.9	181
Allied health professional (therapist, radiographer, assistant)	5	2.8	181
Pharmacist	4	2.2	181
Medical intern	3	1.7	181
Technical staff (laboratory, sterilisation)	2	1.1	181
Certified registered nurse	1	0.6	181
Patient-care technician	1	0.6	181
Administration	1	0.6	181
**Physician grade and certification (physicians only)**			
Consultant	15	21.7	69
Assistant consultant	6	8.7	69
Associate consultant	4	5.8	69
Staff physician	4	5.8	69
Senior resident	15	21.7	69
Junior resident	25	36.2	69
Emergency medicine board-certified	29	41.4	70
**Employment and working pattern**			
Full-time	179	98.9	181
Part-time	1	0.6	181
Temporary or casual	1	0.6	181
Works in shifts	175	96.7	181
Works at some time between 18:00 and 07:00	162	89.5	181
**Experience in the health sector**			
Under 1 year	28	15.5	181
1–5 years	90	49.7	181
6–10 years	20	11.0	181
11–15 years	22	12.2	181
16–20 years	8	4.4	181
Over 20 years	13	7.2	181
**Migration to the country of work**			
Moved from another country	56	30.9	181
Time since moving (respondents who had migrated)			
11 months ago or less	9	16.4	55
1–5 years ago	32	58.2	55
6 years ago or more	14	25.5	55
**Patient contact**			
Interacts with patients or clients during work	175	96.7	181
Routine direct physical contact (washing, turning, lifting)	125	71.4	175
Works mainly with both male and female patients	162	92.6	175
Patient age groups usually cared for †			
Newborns	47	26.0	181
Infants	52	28.7	181

Characteristic	n	%	Denominator (N)
Children	68	37.6	181
Adolescents (10–18 years)	95	52.5	181
Adults	141	77.9	181
Older adults	112	61.9	181
**Work setting**			
Hospital	178	98.3	181
Ambulance	3	1.7	181
Main hospital service (respondents reporting a named service)			
Emergency	160	90.9	176
Specialised unit (paediatrics, orthopaedics, radiology)	6	3.4	176
Support services (kitchen, maintenance)	4	2.3	176
General medicine	2	1.1	176
Intensive care	2	1.1	176
Ambulatory	1	0.6	176
Multiple different rotations	1	0.6	176
**Staff present in the work setting for most of the working time**			
None	3	1.7	181
1–5 staff	52	28.7	181
6–10 staff	46	25.4	181
11–15 staff	57	31.5	181
More than 15 staff	23	12.7	181
**Worry about violence in the current workplace**			
Not worried at all	48	26.5	181
Slightly worried	38	21.0	181
Moderately worried	57	31.5	181
Worried	22	12.2	181
Very worried	16	8.8	181
**Reporting environment**			
Procedures for reporting violence exist in the workplace	163	90.1	181
Knows how to use the procedures	126	77.8	162
Encouraged to report workplace violence	131	72.4	181
Source of encouragement (respondents reporting encouragement) †			
Management or employer	113	86.3	131
Colleagues	60	45.8	131
Own family or friends	21	16.0	131
Society	9	6.9	131
Hospital policies	1	0.8	131

Values are n/N (%). Percentages use the number with non-missing data for the item as denominator. Patient age groups and sources of encouragement are multiple-response items.

*† Multiple responses permitted; percentages need not sum to 100%. Physician** grade was answered by 69 of the 70 physicians. Timing of migration was answered by 55 of the 56 respondents who had migrated. Routine direct physical contact and patient gender were shown only to the 175 respondents reporting interaction with patients. Main hospital service applies to the 176 respondents who reported a hospital or ambulance setting with a named service. Knowledge of the reporting procedures was asked only of the 162 respondents who reported that procedures existed and who answered the follow-up item.*

Table S3. Characteristics, consequences and responses to the most recent physical-violence incident (n = 16)

Domain and item	n (%)
Occurrence	
Physical attack in the previous 12 months	16/181 (8.8)
Considered the incident typical	14 (87.5)
Perpetrator*	
Patient or client	14 (87.5)
Relative of a patient or client	8 (50.0)
Staff member	5 (31.3)
External colleague or worker	2 (12.5)
Management or supervisor	1 (6.3)
Location	
Inside a health institution or facility	13 (81.3)
At a patient's or client's home	2 (12.5)
Outside (en route to work, health visit or home)	1 (6.3)
Time of incident	
07:00 to before 13:00	3 (18.8)
13:00 to before 18:00	3 (18.8)
18:00 to before 24:00	1 (6.3)
24:00 to before 07:00	3 (18.8)
Does not remember	6 (37.5)
Response to the incident*	
Took no action	8 (50.0)
Told the person to stop	7 (43.8)
Told a senior staff member	6 (37.5)
Filed an Occurrence Variance Report	6 (37.5)
Tried to defend self physically	5 (31.3)
Told friends or family	4 (25.0)
Told a colleague	2 (12.5)
Transferred to another position	2 (12.5)
Pretended it never happened	1 (6.3)
Sought counselling	1 (6.3)
Sought help from the union	1 (6.3)
Completed a compensation claim	1 (6.3)
Pursued prosecution	1 (6.3)
Told a senior colleague and/or filed an OVR	10 (62.5)
Preventability, harm and absence	
Considered the incident preventable	11 (68.8)
Injured as a result	6 (37.5)
Required formal treatment for the injury	4 (25.0)
Took time off work	7 (43.8)
Investigation and consequences	
Investigation undertaken: yes	7 (43.8)
Investigation undertaken: no	8 (50.0)
Investigation undertaken: does not know	1 (6.3)
by management or employer*	6 (37.5)

Domain and item	n (%)
by police*	2 (12.5)
by a community group*	1 (6.3)
by a society*	1 (6.3)
No consequence for the attacker*	7 (43.8)
Verbal warning issued*	4 (25.0)
Attacker reported to police*	3 (18.8)
Consequence not known*	4 (25.0)
Employer or supervisor support*	
Offered counselling	8 (50.0)
Offered an opportunity to speak about or report the incident	7 (43.8)
Offered other support	4 (25.0)
Satisfaction with how the incident was handled	
Very dissatisfied	6 (37.5)
Dissatisfied	3 (18.8)
Neutral	4 (25.0)
Satisfied	1 (6.3)
Very satisfied	2 (12.5)
Reasons endorsed for not reporting or telling others*†	
Not important	7 (43.8)
Useless	5 (31.3)
Afraid of negative consequences	5 (31.3)
Felt ashamed	3 (18.8)
Felt guilty	3 (18.8)
Did not know whom to report to	1 (6.3)

Values are n (%). Unless otherwise stated the denominator is the 16 healthcare workers reporting physical attack in the previous 12 months.

** Multiple responses permitted; percentages need not sum to 100%. † These items were displayed to all respondents reporting physical attack rather than only to those who did not report — 10 of the 16 had told a senior colleague or filed an OVR — and should therefore be read as barriers endorsed by attacked respondents in general, not as reasons among non-reporters.*

Table S4. Characteristics, consequences and responses to the most recent verbal-abuse incident (n = 71)

Characteristic	n	%	Denominator (N)
**Frequency of verbal abuse in the previous 12 months**			
Once	12	16.9	71
Sometimes	48	67.6	71
All the time	11	15.5	71
Considered the most recent incident typical of the workplace	56	78.9	71
**Perpetrator of the most recent incident †**			
Relative of a patient or client	43	60.6	71
Patient or client	37	52.1	71
Staff member	16	22.5	71
Management or supervisor	9	12.7	71
External colleague or worker	5	7.0	71
General public	3	4.2	71
**Location of the most recent incident**			
Inside a health institution or facility	66	93.0	71
At a patient's or client's home	3	4.2	71
Outside (en route to work, health visit or home)	2	2.8	71
**Response to the most recent incident †**			
Direct response at the time			
Told the person to stop	31	43.7	71
Called security or the patient relations office	1	1.4	71
Informed security	1	1.4	71
Informal disclosure			
Told a senior staff member	31	43.7	71
Told a colleague	20	28.2	71
Told friends or family	15	21.1	71
Contacted the charge or head nurse	1	1.4	71
Formal or external action			
Completed an incident or accident form	12	16.9	71
Sought help from the union	3	4.2	71
Sought help from an association	2	2.8	71
Pursued prosecution	1	1.4	71
No action or avoidance			
Took no action	24	33.8	71
Pretended it never happened	15	21.1	71
Other action			
Transferred to another position	4	5.6	71
Sought counselling	2	2.8	71
**Preventability and investigation**			
Considered the incident preventable	51	71.8	71
Investigation undertaken: yes	20	28.2	71
Investigation undertaken: no	39	54.9	71
Investigation undertaken: does not know	12	16.9	71
Investigation conducted by (respondents reporting an investigation) †‡			
Management or employer	18	90.0	20
Association or society	2	10.0	20
Community group	2	10.0	20
**Consequences for the abuser (respondents reporting an investigation) †‡**			

Characteristic	n	%	Denominator (N)
Verbal warning issued	12	60.0	20
Reported to police	4	20.0	20
Care discontinued	1	5.0	20
Aggressor prosecuted	1	5.0	20
None	5	25.0	20
Does not know	5	25.0	20
**Support offered by the employer or supervisor †**			
Opportunity to speak about or report the incident	35	49.3	71
Counselling	20	28.2	71
Other support	19	26.8	71
**Satisfaction with how the incident was handled**			
Very dissatisfied	16	22.5	71
Dissatisfied	12	16.9	71
Neutral	27	38.0	71
Satisfied	12	16.9	71
Very satisfied	4	5.6	71
**Dissatisfied or very dissatisfied (combined)**	**28**	**39.4**	**71**
**Barriers to reporting endorsed †§**			
Reporting would be useless	27	38.0	71
The incident was not important	20	28.2	71
Afraid of negative consequences	13	18.3	71
Did not know whom to report to	13	18.3	71
Felt ashamed	7	9.9	71
Felt guilty	5	7.0	71
Believed no action would be taken	1	1.4	71
Considered it typical in the area	1	1.4	71
Advised by a senior or colleague not to report	1	1.4	71

Values are n (%). Unless otherwise stated the denominator is the 71 healthcare workers reporting verbal abuse in the previous 12 months.

† Multiple responses permitted; percentages need not sum to 100%.

‡ These items followed the question on whether an investigation was undertaken and were therefore displayed only to the 20 respondents reporting an investigation; percentages are calculated on that branch denominator rather than on all 71 respondents.

§ These items were displayed to all respondents reporting verbal abuse rather than only to those who did not report the incident. They should therefore be read as barriers to reporting endorsed by verbally abused respondents in general, not as reasons given by non-reporters.

Table S5. Employer policies, workplace measures and reported changes (n = 181)

Employer has** a specific policy on**	No	Yes	Does not know
Health and safety	10 (5.5)	134 (74.0)	37 (20.4)
Physical workplace violence	13 (7.2)	128 (70.7)	40 (22.1)
Verbal abuse	21 (11.6)	111 (61.3)	49 (27.1)
Sexual harassment	18 (9.9)	112 (61.9)	51 (28.2)
Racial harassment	26 (14.4)	89 (49.2)	66 (36.5)
Threats	21 (11.6)	108 (59.7)	52 (28.7)
Bullying or mobbing	23 (12.7)	99 (54.7)	59 (32.6)
**Workplace measure reported to exist***	**n (%)**		
Security measures (guards, alarms, portable telephones)	155 (85.6)		
Restricted public access	82 (45.3)		
Improved surroundings (lighting, noise, cleanliness, privacy)	73 (40.3)		
Patient protocols	73 (40.3)		
Training	65 (35.9)		
Patient screening	58 (32.0)		
Increased staff numbers	47 (26.0)		
Changed shifts or rotas	39 (21.5)		
Reduced periods of working alone	38 (21.0)		
Investment in human-resource development	30 (16.6)		
Special equipment or clothing	29 (16.0)		
Staff check-in procedures	29 (16.0)		
Restricted exchange of money at the workplace	27 (14.9)		
None of these measures	5 (2.8)		
Does not know	1 (0.6)		
**Workplace change in the previous two years***	**n (%)**		
Increased staff numbers	28 (15.5)		
Staff cuts	28 (15.5)		
Restriction of resources	27 (14.9)		
Restructuring or reorganization	21 (11.6)		
Additional resources	13 (7.2)		
Lack of security or other support team	1 (0.6)		
Restricted public access	1 (0.6)		
No changes	49 (27.1)		
Does not know	74 (40.9)		
**Perceived impact of changes on daily work***	**n (%)**		
Work situation for staff worsened	39 (21.5)		
Work situation for staff improved	37 (20.4)		
Situation for patients or clients improved	20 (11.0)		
Situation for patients or clients worsened	15 (8.3)		
Free-text: new emergency-department staff underused	1 (0.6)		
Free-text: both positive and negative effects	1 (0.6)		
None	44 (24.3)		
Does not know	63 (34.8)		

Values are n (%).

** Multiple responses permitted; percentages need not sum to 100%.*

Table S6. Perceived helpfulness of preventive measures, full response distributions (n = 181)

Measure	Not at all	Little	Moderate	Very	Mean ± SD
Security measures	11 (6.1)	10 (5.5)	31 (17.1)	129 (71.3)	2.54 ± 0.85
Improve surroundings	10 (5.5)	18 (9.9)	49 (27.1)	104 (57.5)	2.36 ± 0.88
Restrict public access	10 (5.5)	23 (12.7)	42 (23.2)	106 (58.6)	2.35 ± 0.90
Training	9 (5.0)	21 (11.6)	57 (31.5)	94 (51.9)	2.30 ± 0.86
Patient protocols	10 (5.5)	25 (13.8)	54 (29.8)	92 (50.8)	2.26 ± 0.90
Patient screening	11 (6.1)	23 (12.7)	60 (33.1)	87 (48.1)	2.23 ± 0.90
Reduced periods of working alone	13 (7.2)	25 (13.8)	58 (32.0)	85 (47.0)	2.19 ± 0.93
Human resource development	13 (7.2)	23 (12.7)	67 (37.0)	78 (43.1)	2.16 ± 0.91
Increased staff numbers	14 (7.7)	27 (14.9)	59 (32.6)	81 (44.8)	2.14 ± 0.94
Check-in procedures for staff	15 (8.3)	31 (17.1)	55 (30.4)	80 (44.2)	2.10 ± 0.97
Special equipment or clothing	22 (12.2)	41 (22.7)	50 (27.6)	68 (37.6)	1.91 ± 1.04
Changed shifts or rotas	29 (16.0)	31 (17.1)	63 (34.8)	58 (32.0)	1.83 ± 1.05
Restrict exchange of money at the workplace	29 (16.0)	49 (27.1)	45 (24.9)	58 (32.0)	1.73 ± 1.08

Values are n (%). Responses were coded 0 = not at all helpful, 1 = little, 2 = moderate, 3 = very helpful. Measures are ranked by mean rating, as in Table 6.

Table S7. Exposure by covariate category in the analytic sample (n = 175)

The outcome is exposure to physical attack and/or verbal abuse in the previous 12 months.

Covariate	Category	n	Events	Exposed (%)
**Gender**	Male	81	32	39.5
	Female	94	42	44.7
**Professional group**	Physician	70	34	48.6
	Nurse	87	36	41.4
	Other	18	4	22.2
**Work experience**	≤5 years	115	47	40.9
	6–10 years	17	6	35.3
	>10 years	43	21	48.8
**Night-shift work**	No	18	6	33.3
	Yes	157	68	43.3
**Routine direct physical contact**	No	50	15	30.0
	Yes	125	59	47.2
**Encouragement to report**	No	49	24	49.0
	Yes	126	50	39.7

The smallest cell contains four outcome events (the "Other" professional group), which is the principal source of imprecision in the professional-group estimates. Routine direct physical contact was reported by 32/70 (45.7%) physicians, 82/87 (94.3%) nurses and 11/18 (61.1%) other professionals ($\chi^2(2) = 45.83$, $p < 0.001$).

Across all four specifications (Tables S8–S11), the direction, magnitude and statistical significance of the professional-group and routine-contact associations were consistent with the primary model. Routine direct physical contact was associated with an approximately three- to fourfold increase in adjusted odds of exposure in every specification, including the Firth penalized model. Nurses and staff in the combined "Other" group had lower adjusted odds of exposure than physicians in every specification. These professional-group estimates are conditional contrasts among respondents alike on the remaining model variables. Because the groups differ markedly in routine direct physical contact, and because the "Other" category is occupationally heterogeneous and contains only four outcome events, they should not be read as indicating that physicians are at greater risk of workplace violence than nurses in general; see Section 4.

Table S8. Main model, complete-case sample (n = 175; 74 events).

Table S8 reports the primary multivariable model at the coefficient level. Three further analyses test its robustness.

Variable	b	SE	z	P>|z|	OR (95% CI)
Constant	−0.87	0.70	−1.26	0.209	—
Female	0.41	0.36	1.13	0.258	1.50 (0.74, 3.03)
Nurse	−0.98	0.46	−2.16	0.031	0.37 (0.15, 0.91)
Other	−1.50	0.66	−2.27	0.023	0.22 (0.06, 0.82)
6–10 years	−0.06	0.56	−0.11	0.910	0.94 (0.31, 2.82)
>10 years	0.48	0.38	1.26	0.209	1.62 (0.76, 3.44)
Night shift	0.21	0.56	0.38	0.705	1.23 (0.41, 3.68)
Routine contact	1.19	0.45	2.64	0.008	3.27 (1.36, 7.89)
Encouraged	−0.27	0.38	−0.73	0.466	0.76 (0.36, 1.59)

b, log-odds coefficient; SE, Wald standard error; OR, odds ratio; CI, confidence interval (Wald, on the log-odds scale, exponentiated). Reference categories: male, physician, ≤5 years, no night shift, no routine contact, not encouraged to report. Model fit: log-likelihood = −111.199; likelihood-ratio $\chi^2(8) = 16.02$, p = 0.042; McFadden pseudo-R^2 = 0.067; Cox and Snell R^2 = 0.088; Nagelkerke R^2 = 0.118. Discrimination: AUC = 0.657 (95% CI 0.576–0.738). Calibration: Hosmer–Lemeshow $\chi^2(7) = 0.59$, p = 0.999. Variance inflation factors ranged from 1.06 to 1.95. This table reports the same model as the multivariable column of Table 7, expressed at the coefficient level.

Table S9. Sensitivity analysis excluding 9 inconsistent respondents (n = 172; 74 events).

Table S9 excludes the nine respondents whose answers were internally inconsistent with the stated eligibility criteria for the survey.

Variable	β	SE	z	p	Odds ratio (95% CI)
Intercept	−0.965	0.704	−1.37	0.171	—
Female (ref: male)	0.468	0.365	1.28	0.200	1.60 (0.78–3.26)
Nurse (ref: physician)	−1.173	0.476	−2.46	0.014	0.31 (0.12–0.79)
Other professional group (ref: physician)	−1.652	0.673	−2.45	0.014	0.19 (0.05–0.72)
6–10 years (ref: ≤5 years)	−0.107	0.566	−0.19	0.850	0.90 (0.30–2.72)
>10 years (ref: ≤5 years)	0.507	0.391	1.30	0.195	1.66 (0.77–3.57)
Night-shift work, 18:00–07:00 (ref: no)	0.242	0.561	0.43	0.666	1.27 (0.42–3.82)
Routine direct physical contact with patients (ref: no)	1.336	0.464	2.88	0.004	3.80 (1.53–9.44)
Encouragement to report violence (ref: no)	−0.172	0.382	−0.45	0.653	0.84 (0.40–1.78)

Excludes the six respondents who reported no routine interaction with patients or clients and the three who answered "No" to the KKUH site-eligibility item; all nine reported characteristics inconsistent with the study's stated eligibility criteria. b, log-odds coefficient; SE, Wald standard error; OR, odds ratio; CI, confidence interval. Log-likelihood = −108.367; likelihood-ratio $\chi^2(8) = 18.35$, p = 0.019. Discrimination: AUC = 0.673. Estimates are materially unchanged from Table S8; the routine-contact association strengthens slightly (adjusted OR 3.80 versus 3.27).

Table S10. Sensitivity analysis with missing routine-contact responses recoded to "No" (n = 181; 74 events).

Table S10 recodes the six structurally missing responses on routine direct physical contact as "No" and refits the model on the full sample of 181 respondents.

**Variable**	**b**	**SE**	**z**	**P>∣z∣**	**OR (95% CI)**
Constant	−0.90	0.69	−1.29	0.197	0.41 (0.10, 1.59)
Female	0.42	0.36	1.19	0.236	1.53 (0.76, 3.09)
Nurse	−1.02	0.45	−2.25	0.024	0.36 (0.15, 0.88)
Other	−1.67	0.64	−2.62	0.009	0.19 (0.05, 0.66)
6–10 years	−0.10	0.56	−0.18	0.855	0.90 (0.30, 2.69)
>10 years	0.49	0.38	1.28	0.200	1.64 (0.77, 3.48)
Night shift	0.20	0.56	0.37	0.714	1.23 (0.41, 3.65)
Routine contact	1.25	0.45	2.79	0.005	3.48 (1.45, 8.35)
Encouraged	−0.28	0.38	−0.74	0.458	0.76 (0.36, 1.58)

The six respondents who were not shown the routine-contact item because of the questionnaire's skip logic (no interaction with patients or clients) are recoded as "No" on this item and included, giving the full study sample. b, log-odds coefficient; SE, Wald standard error; OR, odds ratio; CI, confidence interval. Log-likelihood = −111.680; likelihood-ratio $\chi^2(8) = 21.51$, $p = 0.006$. Estimates are essentially unchanged from Table S8. Complete-case analysis was retained for the main results because recoding a structurally missing response as a measured negative alters the meaning of the variable.

Table S11. Firth penalized-likelihood logistic regression, complete-case sample (n = 175; 74 events).

Variable	b	SE	z	P>|z|	OR	Wald 95% CI	Profile PL 95% CI
Constant	−0.80	0.69	−1.16	0.247	0.45	(0.12, 1.74)	—
Female	0.38	0.36	1.08	0.282	1.47	(0.73, 2.95)	(0.74, 2.94)
Nurse	−0.93	0.45	−2.06	0.040	0.40	(0.16, 0.96)	(0.16, 0.93)
Other	−1.36	0.64	−2.12	0.034	0.26	(0.07, 0.90)	(0.07, 0.81)
6–10 years	−0.04	0.56	−0.07	0.943	0.96	(0.32, 2.86)	(0.32, 2.71)
>10 years	0.46	0.38	1.21	0.225	1.59	(0.75, 3.36)	(0.76, 3.33)
Night shift	0.17	0.55	0.32	0.753	1.19	(0.40, 3.50)	(0.43, 3.56)
Routine contact	1.11	0.44	2.51	0.012	3.04	(1.28, 7.22)	(1.33, 7.34)
Encouraged	−0.27	0.37	−0.71	0.476	0.77	(0.37, 1.59)	(0.38, 1.57)

Firth's bias-reduced (penalized-likelihood) logistic regression. b, penalized-likelihood coefficient; SE, Wald standard error from the penalized-likelihood information matrix; OR, odds ratio; Wald 95% CI, conventional symmetric CI on the log-odds scale; Profile PL 95% CI, penalized profile-likelihood confidence interval. A profile-likelihood interval is not reported for the constant. Statistical significance should be read from the penalized profile-likelihood intervals, which are the intervals quoted in the text. Estimates are attenuated toward the null relative to Table S8, most markedly for the "Other" professional group, as expected given its four outcome events; the direction and statistical interpretation of every estimate are unchanged.

Supplementary File S1. Administered questionnaire

Adapted from the ILO/ICN/WHO/PSI Joint Programme on Workplace Violence in the Health Sector, *Workplace Violence in the Health Sector Country Case Study Questionnaire* (Geneva, 2003). Administered in English via an anonymous online form with conditional skip logic. The incident-specific modules on bullying/mobbing (C.II), sexual harassment (C.III), and racial harassment (C.IV) were not administered. Items marked * were required.

**Participant information and consent

Participants were shown a study information page describing the purpose of the study, voluntary participation, the right to withdraw without consequence, anonymity and confidentiality of responses, and contact details for the principal investigator. Consent was recorded by a required item: "I agree to participate in this study and acknowledge that my responses will remain anonymous" / "I do not agree to participate in this study". An administrative item recorded the data collector number (1–8).

**Section A. Personal and workplace data

1. Sex*: male; female
2. Age*: <30; 30–39; 40–49; 50+
3. Marital status*: single; married; separated/divorced; widow/widower
4. Nationality*: Saudi; non-Saudi
5. Department*: emergency department; outpatient; inpatient
6. Present professional group*: physician; nurse; ambulance; pharmacist; administration; professions allied to medicine (therapists/radiographers/assistants); support staff (kitchen/maintenance, security); technical staff (laboratory/sterilisation); other
7. If physician, type of physician: junior resident; senior resident; staff physician; associate consultant; assistant consultant; consultant
8. Emergency medicine board certification*: yes; no; other
9. Did you move from another country to the place where you are currently working?*: yes; no
10. If yes, when did you move?: 11 months ago or less; 1–5 years ago; 6 years ago or more
11. Please identify how you see yourself within each of the following settings* (in the country; in the community; at your workplace): member of the majority ethnic group; member of the minority ethnic group
12. Years of work experience in the health sector*: under 1 year; 1–5 years; 6–10 years; 11–15 years; 16–20 years; over 20 years
13. Do you work at KKUH?*: yes; no; other
14. In your main job, do you work*: full-time; part-time; temporary/casual
15. Do you work in shifts?*: yes; no
16. Do you work anytime between 18:00 and 07:00?*: yes; no
17. Do you interact with patients/clients during your work?*: yes; no

**Patient interaction (shown only to those answering yes to item 17)

18. Do you have routine direct physical contact (washing, turning, lifting) with patients/clients?*: yes; no
19. The patients/clients you most frequently work with are (tick all that apply)*: newborn; infants; children; adolescents (10–18); adults; elderly

20. The gender of the patients you most frequently work with*: male; female; both

**Workplace

21. Do you spend more than 50% of your time working with any of the following specialities?*: physically disabled; mentally disabled; home care; terminally ill; HIV/AIDS; psychiatric; mother/child care; geriatric; occupational health and safety; school health; none; other
22. Where do you spend most of your time (more than 50%) in your main job?*: hospital; ambulance; health centre; community/district; hospice; home for the elderly/nursing home; rehabilitation centre/convalescent home; other
23. If hospital, the main service being: emergency; ambulatory; general surgery; general medicine; operating room; psychiatric; intensive care; management; specialised unit; technical services; support services; other
24. Number of staff present in the same work setting during most of your work time*: none; 1–5; 6–10; 11–15; over 15
25. How worried are you about violence in your current workplace?* (1 = not worried at all to 5 = very worried)
26. Are there procedures for the reporting of violence in your workplace?*: yes; no
27. If yes, do you know how to use them?: yes; no
28. Is there encouragement to report workplace violence?*: yes; no
29. If yes, by whom? (tick all that apply): management/employer; colleagues; own family/friends; society; other

**Section B. Physical workplace violence

30. In the last 12 months, have you been physically attacked in your workplace?*: yes; no

Items 31–47 were shown only to respondents answering yes to item 30.

31. How would you describe the last incident of physical violence in your workplace?*: physical violence without a weapon; physical violence with a weapon
32. Do you consider this a typical incident of violence in your workplace?*: yes; no
33. Who attacked you? (tick all that apply)*: patient/client; relatives of patient/client; staff member; management/supervisor; external colleague/worker; general public; other
34. Where did the incident take place?*: inside health institution or facility; at patient's/client's home; outside (on way to work/health visit/home)
35. At what time did it happen?*: 07:00–before 13:00; 13:00–before 18:00; 18:00–before 24:00; 24:00–before 07:00; don't remember
36. On which day of the week did it happen?*: Sunday through Saturday; don't remember
37. How did you respond to the incident? (tick all that apply)*: took no action; told the person to stop; incident report (OVR); tried to pretend it never happened; tried to defend myself physically; told friends/family; sought counselling; told a colleague; reported it to a senior staff member; transferred to another position; sought help from an association; sought help from the union; pursued prosecution; completed a compensation claim; other
38. Do you think the incident could have been prevented?*: yes; no
39. Were you injured as a result of the violent incident?*: yes; no
40. If yes, did you require formal treatment for the injuries?: yes; no

41. How bothered have you been by these experiences since the attack?* Five items, each rated not at all / a little bit / moderately / quite a bit / extremely: overall level of being bothered; repeated disturbing memories, thoughts or images of the attack; avoiding thinking or talking about the attack, or avoiding related feelings; being "super-alert" or watchful and on guard; feeling that everything you did was an effort
42. Did you have to take time off from work after being attacked?*: yes; no
43. If yes, for how long: one day; 2–3 days; one week; 2–3 weeks; 1 month; 2–6 months; 7–12 months
44. Was any action taken to investigate the causes of the incident?*: yes; no; don't know
45. If yes, by whom? (tick all that apply): management/employer; society; community group; police; other
46. What were the consequences for the attacker? (tick all that apply)*: none; verbal warning issued; care discontinued; reported to police; aggressor prosecuted; don't know; other
47. Did your employer or supervisor offer to provide you with* (counselling; opportunity to speak about/report it; other support): yes; no
48. How satisfied are you with the manner in which the incident was handled?* (1 = very dissatisfied to 5 = very satisfied)
49. If you did not report or tell about the incident to others, why not? (tick all that apply): it was not important; felt ashamed; felt guilty; afraid of negative consequences; useless; did not know who to report to; other

**Witnessed physical violence and reporting

50. In the last 12 months, have you witnessed incidents of physical violence in your workplace?*: yes; no
51. If yes, how often has this occurred in the last 12 months?: once; 2–4 times; 5–10 times; several times a month; about once a week; daily
52. Have you reported an incident of workplace violence in the last 12 months (witnessed or experienced)?*: yes; no
53. If yes, have you been disciplined for reporting an incident of workplace violence?: yes; no

**Section C.I. Psychological workplace violence (verbal abuse)

54. In the last 12 months, have you been verbally abused in your workplace?*: yes; no

Items 55–66 were shown only to respondents answering yes to item 54.

55. How often have you been verbally abused in the last 12 months?*: all the time; sometimes; once
56. Thinking of the last time you were verbally abused, who verbally abused you? (tick all that apply)*: patient/client; relatives of patient/client; staff member; management/supervisor; external colleague/worker; general public; other
57. Do you consider this to be a typical incident of verbal abuse in your workplace?*: yes; no; other
58. Where did the verbal abuse take place?*: inside health institution or facility; at patient's/client's home; outside (on way to work/health visit/home); other
59. How did you respond to the verbal abuse? (tick all that apply)*: took no action; tried to pretend it never happened; told the person to stop; told friends/family; told a colleague; reported it to a senior staff member; sought counselling; sought help from the union; sought help from the association; transferred to another position; completed incident/accident form; pursued prosecution; completed a compensation claim; other

60. How bothered have you been by these experiences since you were abused?* Five items, each rated not at all / a little bit / moderately / quite a bit / extremely, worded as in item 41 with reference to the abuse
61. Do you think the incident could have been prevented?*: yes; no
62. Was any action taken to investigate the causes of the verbal abuse?*: yes; no; don't know
63. If yes, by whom was the action taken? (tick all that apply): management/employer; association (society); community group
64. If yes, what were the consequences for the abuser? (tick all that apply): none; verbal warning issued; care discontinued; reported to police; aggressor prosecuted; don't know; other
65. Did your employer or supervisor offer to provide you with* (counselling; opportunity to speak about/report it; other support): yes; no
66. How satisfied are you with the manner in which the incident was handled?* (1 = very dissatisfied to 5 = very satisfied)
67. If you did not report or tell about the incident to others, why not? (tick all that apply): it was not important; felt ashamed; felt guilty; afraid of negative consequences; did not know who to report to; useless; other

**Section D. Health sector employer

68. Has your employer developed specific policies on* (health and safety; physical workplace violence; verbal abuse; sexual harassment; racial harassment; bullying/mobbing; threat): yes; no; don't know
69. What measures to deal with workplace violence exist in your workplace? (tick all that apply)*: security measures (guards, alarms, portable telephones); improve surroundings (lighting, noise, heat, access to food, cleanliness, privacy); restrict public access; patient screening; patient protocols; restrict exchange of money at the workplace; increased staff numbers; check-in procedures for staff; special equipment or clothing; changed shifts or rotas; reduced periods of working alone; training; investment in human resource development; none of these; other
70. To what extent do you think these measures would be helpful in your work setting?* Thirteen measures, each rated very / moderate / little / not at all: security measures; improve surroundings; restrict public access; patient screening; patient protocols; restrict exchange of money at the workplace; increased staff numbers; check-in procedures for staff; special equipment or clothing; changed shifts or rotas; reduced periods of working alone; training; human resource development
71. Which of the following changes, if any, have occurred in the workplace or health care setting in the last 2 years? (tick all that apply)*: none; staff cuts; restriction of resources; restructuring/reorganization; increased staff numbers; additional resources; don't know; other
72. In your opinion, what impact have the above changes had on your daily work? (tick all that apply)*: none; work situation for staff worsened; work situation for staff improved; situation for patients/clients worsened; situation for patients/clients improved; don't know; other

**Optional closing items

Two optional free-text fields for a name and a mobile number appeared on the final page. No respondent completed either field, and neither was included in the analytic dataset or used in any analysis.
